# Genetic Analysis of the Peach SnRK1β3 Subunit and Its Function in Transgenic Tomato Plants

**DOI:** 10.3390/genes15121574

**Published:** 2024-12-06

**Authors:** Shilong Zhao, Xuelian Wu, Jiahui Liang, Zhe Wang, Shihao Fan, Hao Du, Haixiang Yu, Yuansong Xiao, Futian Peng

**Affiliations:** College of Horticulture Science and Engineering, Shandong Agricultural University, Tai’an 271018, China; zslintaian@126.com (S.Z.);

**Keywords:** peach, PpSnRK1β3, transgenic tomato plants

## Abstract

Background/Objectives: The sucrose non-fermentation-related kinase 1 (SnRK1) protein complex in plants plays an important role in energy metabolism, anabolism, growth, and stress resistance. SnRK1 is a heterotrimeric complex. The SnRK1 complex is mainly composed of α, β, βγ, and γ subunits. Studies on plant SnRK1 have primarily focused on the functional α subunit, with the β regulatory subunit remaining relatively unexplored. The present study aimed to elucidate the evolutionary relationship, structural prediction, and interaction with the core α subunit of peach SnRK1β3 (PpSnRK1) subunit. Methods: Bioinformatics analysis of PpSnRK1 was performed through software and website. We produced transgenic tomato plants overexpressing PpSnRK1 (OEPpSnRK1). Transcriptome analysis was performed on OEPpSnRK1 tomatoes. We mainly tested the growth index and drought resistance of transgenic tomato plants. Results: The results showed that PpSnRK1 has a 354 bp encoded protein sequence (cds), which is mainly located in the nucleus and cell membrane. Phylogenetic tree analysis showed that PpSnRK1β3 has similar domains to other woody plants. Transcriptome analysis of OEPpSnRK1β3 showed that PpSnRK1β3 is widely involved in biosynthetic and metabolic processes. Functional analyses of these transgenic plants revealed prolonged growth periods, enhanced growth potential, improved photosynthetic activity, and superior drought stress tolerance. Conclusions: The study findings provide insight into the function of the PpSnRK1 subunit and its potential role in regulating plant growth and drought responses. This comprehensive analysis of PpSnRK1 will contribute to further enhancing our understanding of the plant SnRK1 protein complex.

## 1. Introduction

Sucrose non-fermentation-associated kinases 1 (SnRK1s) are a conserved family of protein kinases eukaryotes, including plants. They play an important role in energy and metabolic homeostasis. In plants, SnRK1s encode a larger subfamily with unique features that are distinct from their counterparts in yeast and mammals [1]. They share homology with the sucrose non-fermenting 1 (SNF1) kinase in yeast and the AMP-activated protein kinase (AMPK) in mammals [2,3]. SnRK1 is a heterotrimeric complex. The SnRK1 family comprises catalytic α subunits and non-catalytic β, βγ, and γ subunits. The *Arabidopsis* genome encodes homologs for each subunit, including two atypical subunits, β3 and βγ, which contain a unique domain structure found exclusively in plants. This diverse SnRK1 subfamily plays an important role in maintaining metabolic homeostasis and adapting to various environmental conditions in plants [4].

SnRK1 is a central protein in coordinating energy use, metabolic processes, stress responses, and growth in plants, which indicates its diverse and important roles in plant physiology [5]. Trehalose-6-phosphate, which is a key intermediate in trehalose metabolism, plays a significant role in controlling embryonic and vegetative growth, flowering time, and meristem fate in plants. Studies indicate that trehalose-6-phosphate interacts with members of the SnRK1 family, which act as energy sensors and regulate plant growth during metabolic stress to maintain energy equilibrium [6,7]. In addition to its role in energy sensing, SnRK1 regulates sugar metabolism and starch biosynthesis. Transgenic tobacco plants expressing StSnRK1 show a significant increase in starch, sucrose, glucose, and fructose content, highlighting the importance of SnRK1 in metabolic processes [8]. SnRK1 directly phosphorylates key metabolic enzymes and regulatory proteins, participates in extensive transcriptional regulation, and downregulates TOR kinase signaling [9]. Moreover, it enhances stress tolerance in plants and contributes to improved abiotic stress tolerance in tomato plants [10]. In addition, *Arabidopsis thaliana* SnRK1 can sense changes in energy levels and activate autophagy, a cellular degradation process that enables cells to recycle their contents during environmental stress or cell remodeling [11].

The β subunit serves as the structural core of the SnRK1 kinase complex and connects the α and γ subunits. The β subunit harbors two distinct domains: a GBD domain and an ASC domain, which primarily regulate the interaction with the SnRK1γ subunit in plants [12,13]. The β subunit in yeast may also facilitate the interaction between the kinase and its target protein through its non-conserved N-terminal region. Furthermore, the N-terminal and GBD domains of the β subunit may control the subcellular localization of the kinase complex [14]. Studies on the β subunit of the SnRK1 complex in plants revealed that a reduction in StubGAL83 (β subunit) expression results in abnormal root and tuber development in potatoes, indicating its importance in plant growth and development [15]. In addition, it was observed that the SnRK1β subunit, which is a target gene of N-myristoyltransferase, an important gene for plant embryonic development, affects plant shoot and root growth by altering cell localization [16]. Moreover, the expression of the SnRK1β subunit in the leaves of *A. thaliana* significantly changes in a salt environment, suggesting that the β subunit responds to extracellular environmental cues similar to the core subunit [17,18].

The SnRK1β3 subunit is unique to plants. It differs from other β subunits because of its truncated nature, lacking both the CBM domain and the N-terminal region [9,19]. Studies suggest that SnRK1β3 serves as a connector between the α and γ subunits, which stabilizes the structure of the SnRK1 complex [20,21]. This subunit regulates the activity of the entire complex by interacting with the SnRK1βγ subunit [22]. Immunostaining revealed that SnRK1β3 is abundantly present in chloroplasts, particularly in starch granules, suggesting its involvement in polysaccharide metabolism [23]. Notably, the expression of SnRK1β3 is relatively high in plant flowers compared with other subunits [24]. Furthermore, the SnRK1β3 subunit interacts with various FCS-like zinc finger (FLZ) proteins, thus hinting at a potential role in plant energy homeostasis [25].

Peach [*Prunus persica* (L.) Batsch], a globally significant deciduous fruit crop, has immense economic and nutritional value [26]. Functional studies on peach SnRK1 (PpSnRK1) have been conducted. In the PpSnRK1 overexpression line, genes related to coenzyme synthesis and transport are significantly upregulated, allowing them to respond to changes in exogenous sucrose concentrations [27]. Overexpression of PpSnRK1α boosts the transcriptional activity of the leucine zipper transcription factor 11 (bZIP11), an important regulator of trehalose metabolism. This enhances trehalose metabolism and safeguards plants against trehalose-induced damage [28]. Furthermore, PpSnRK1 stimulates sorbitol metabolism and elevates sucrose accumulation in the peach fruit, which may enhance fruit quality [29]; however, the structure and function of the PpSnRK1β3 subunit remains unclear.

In this study, the structure, subcellular localization, and genetic evolution of PpSnRK1β3 were analyzed, validating its relationship with the α core subunit of SnRK1. Functional verification of PpSnRK1β3 overexpressed in tomato demonstrated its extensive involvement in peach biology, in which it enhances chlorophyll synthesis, photosynthesis, and drought tolerance. This furthers our understanding of SnRK1 subunit function and may lead to genetic improvement strategies in plants.

## 2. Materials and Methods

### 2.1. Identification of the PpSnRK1β3 Subunit

Protein and gene sequences for the SnRK1 genes in *P. persica* were obtained from the NCBI (National Center for Biotechnology Information). The PpSnRK1β3 gene was amplified using 25 μL of amplification enzyme 2 × Phanta Max Master Mix (Dye Plus) (Vazyme, Nanjing, China), 18 μL ddH2O, 4 μL of PpSnRK1β3 homologous recombination upstream/downstream primers (Primers:Table S2) and 3 μL of cDNA from peach seedling leaf. The pRI-101-GFP plasmid was used as a vector [30]. Tertiary structure prediction of PpSnRK1 was performed (https://swissmodel.expasy.org/interactive, accessed on 16 May 2024) using the SWISS-MODEL website. The model with the highest matching degree was selected [31].

### 2.2. Subcellular Localization of PpSnRK1β3

The PpSnRK1β3 cds was cloned into the pRI101-GFP vector (Primers: Table S2). This vector was then introduced into *Agrobacterium* (GV3101) and injected into Tobacco benthamiana leaves with an OD value adjusted to 0.6–0.8. Subsequently, the position of the GFP protein within the tobacco cells was visualized by laser confocal microscopy (LSM880, Zeiss, Jena, Germany) following a 2-day dark treatment [32]. A mixed field displaying the GFP fluorescence and bright-field signals were obtained to indicate the precise subcellular localization of PpSnRK1β3 within the plant cells.

### 2.3. Phylogenetic Tree Analysis

Protein sequences of SnRK1β3 were gathered from the NCBI database for 14 plant species (https://www.ncbi.nlm.nih.gov/, accessed on 18 May 2024), which included *A. thaliana*, *Populus trichocarpa*, *P. persica*, *Vitis vinifera*, *Malus domestica*, *Prunus avium*, *Ananas comosus*, *Nicotiana attenuata*, *Carya illinoinensis*, *Durio zibethinus*, *Mangifera indica*, *Pyrus x bretschneideri*, *Triticum aestivum*, and *Gossypium hirsutum*. These sequences were analyzed using MEGA (MEGA7) software to construct an evolutionary tree using the neighbor-joining method with 1000 bootstrap copies [33]. Structural domains were analyzed using Pfam (http://pfam-legacy.xfam.org/, accessed on 18 May 2024) [34]. Protein sequence alignment was performed using DNAMAN (v9.0) software [35].

### 2.4. Yeast Two-Hybrid (Y2H) Assay

The PpSnRK1α cds was cloned into the PGBKT7 vector, and the cds of PpSnRK1β3 was cloned into the PGADT7 vector (Primers: Table S2). These two vectors were transformed together into Y2H gold yeast strain and cultured on SD/–T-L (–Leu/–Trp) and SD/–T-L-H-A (–Leu/–Trp/-His/–Ade) selective medium and incubated at 28 °C for two days. The strong interaction genes PGBKT7-53 and PGADT7-T were transformed as positive controls, and PGBKT7-α and PGADT7 empty vectors, PGADT7-β3 and PGBKT7 empty vectors, and two empty vectors were used as negative controls [36]. The yeast spots on the two-defect medium were diluted with ddH_2_O by 10^−1^, 10^−2^, and 10^−3^ fold to the four-defect medium to observe the growth of the yeast.

### 2.5. Bimolecular Fluorescence Complementation (BiFC) Assay

The cds of PpSnRK1α was cloned into the YC vector and PpSnRK1β3 was cloned into the YN vector (Primers: Table S2). The two vectors were mixed, transferred to *Agrobacterium* GV3101, and injected into the leaves of Nicotiana benthamiana. The PpSnRK1α-YC with YN empty vectors and PpSnRK1β3-YN with YC empty vectors were used as negative controls [37]. Fluorescence within the tobacco cells was observed using laser confocal microscopy (LSM880, Zeiss, Germany) after two days of dark treatment. DAPI indicated the location of the nucleus, and the mixed field was composed of a bright field, YFP fluorescence field, and DAPI field.

### 2.6. Dual Luciferase Assay

The coding sequences of PpSnRK1α and PpSnRK1β3 were cloned into the pGreenII 0800-nLUC vector and the pGreenII 0800-cLUC vector (Primers: Table S2). Negative controls included the PpSnRK1β3-CLUC and NLUC empty vectors, as well as the PpSnRK1α-NLUC and CLUC empty vectors, along with the empty NLUC and CLUC vectors. These vectors were introduced into *Agrobacterium* GV3101 and delivered into the leaves of Nicotiana benthamiana. Following a 3-day incubation period, fluorescence was visualized using a fluorescence microscope (AXIO, Zeiss, Germany) [36].

### 2.7. Acquisition and Experimental Treatment of Overexpressed Tomato Material

The cds of PpSnRK1β3 was cloned into the Pri101 vector and transformed into *Agrobacterium* GV3101. Based on the method of Goel D et al. [38], the stem segments of tomatoes were infected with *Agrobacterium*, and the transgenic T0-generation plants were obtained in symbiotic medium, differentiation medium, and rooting medium (Figure S1a). Screening began at the T0 stage, where plants were grown in normal soil media for further selection. After two generations of screening and typing, the T2 generation of overexpressing PpSnRK1β3-1, PpSnRK1β3-2, and PpSnRK1β3-3 strains was obtained (Figure S1b,c). According to the method described by Fan, the gene expression of PpSnRK1β3 in T2-generation tomatoes was verified by qRT-PCR [39]. The EF-1α gene of tomatoes was used as the internal reference gene [40]. Primers for RT-PCR and qRT-PCR are listed in Table S2.

In 2024, the experiments were conducted at Shandong Agricultural University’s experimental base in Tai’an City, Shandong Province, China (36°170′745,9″ N, 117°16′771,2″ E). Each T2-generation tomato was planted in a plastic black square 8.5 cm × 6 cm × 8 cm pot, mixed with the substrate at a peat soil:vermiculite = ratio of 1:1, and cultivated in the tissue culture laboratory under light:darkness = 16 h:8 h conditions.

Transgenic and wild-type tomatoes with consistent growth at the seedling stage were selected for transcriptome sequencing and the determination of physiological indicators. Transgenic and wild-type tomatoes were treated with 4% PEG-6000 to simulate a drought environment for 14 days, and the tomatoes without drought treatment were used as the control [41]. Physiological indicators of the tomatoes under stress in each group were measured.

### 2.8. GO and KEGG Enrichment Analysis of Differentially Expressed Genes

The transcription and sequencing work was completed by the Nanjing Jisihuiyuan Company (Nanjing, China). The clean reads underwent extensive bioinformatics analysis. Alignment to the reference genome was achieved using HISAT2 software (v2.2.1), whereas RSEM was used for quantifying gene abundance. Genes with a fold change ≥ 1 and *p*-values ≤ 0.05 were considered differentially expressed genes (DEGs) using DEGseq2 (v1.46) [42]. Gene Ontology (GO) terms were considered significantly enriched if they had corrected *p*-values ≤ 0.05. Kyoto Encyclopedia of Genes and Genomes (KEGG) analysis was used to identify DEGs that were significantly enriched in metabolic pathways at *p* ≤ 0.05 using the clusterProfiler R package (v4.14.3) [43].

### 2.9. Determination of Chlorophyll Content

Samples of fresh, clean tomato leaves (0.2 g) were extracted for 24 h in a 95% ethanol solution. The extract was analyzed using a Pharma-Spec UC-2450 ultraviolet spectrophotometer from Shimadzu (Kyoto, Japan) at OD665, OD649, and OD470. These measurements were used to calculate the chlorophyll content of the leaves [44].

### 2.10. Determination of Photosynthetic Parameters

The net photosynthetic rate (Pn) was recorded using a CIRAS-3 portable photosynthetic system (CIRAS-3, PP Systems, Amesbury, MA, USA) under light conditions [43]. A SPAD chlorophyll instrument (spad-502, Hanshatech, Taian, China) was used to determine the leaf SPAD value [45]. The leaves were darkened for 30 min, and then the Fv/Fm ratio was determined using a hand-held leaf fluorometer (Handy PEA, Hanshatech, Taian, China) [46].

### 2.11. Measurement of MDA, H_2_O_2_, O_2_^−^, Relative Electrolyte Leakage

The malondialdehyde (MDA) content of the tomatoes was measured using the thiobarbituric acid (TBA) method [47]. The relative electrolyte leakage of the tomatoes was assessed using a DDS-12 conductometer (Hangzhou Wanda Instrument Factory, Hangzhou, China) [48]. In addition, the hydrogen peroxide content in the tomato leaves was determined by the trichloroacetic acid (TCA) method, and the superoxide anion content of the tomato leaves was measured using the sulfonamide colorimetric method [49].

## 3. Results

### 3.1. Gene Length, Structure Prediction, and Subcellular Localization of PpSnRK1β3

**Figure 1:**
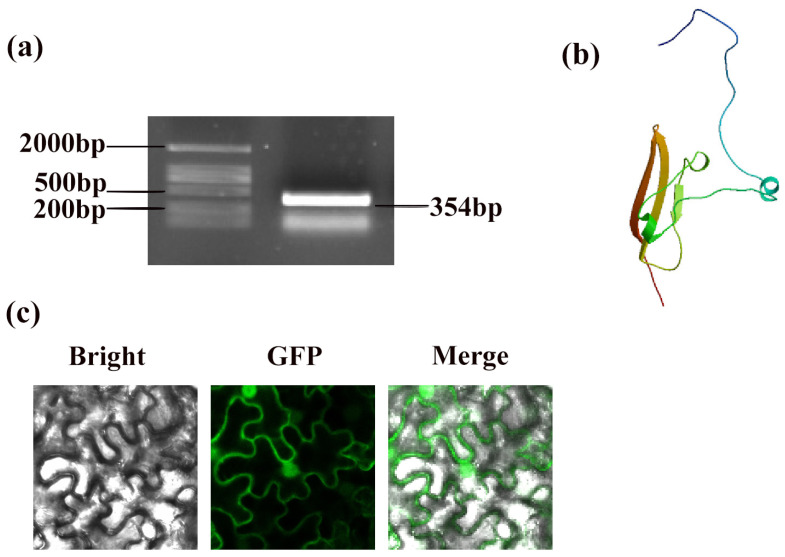
Length, structure prediction, and subcellular localization of PpSnRK1β3. (**a**) The cds length electropherogram of PpSnRK1β3. (**b**) Spatial structure prediction of PpSnRK1β3. (**c**) Subcellular localization of PpSnRK1β3.

The genetic sequence of PpSnRK1β3 (Prupe.6G107300) was retrieved from the NCBI database (https://www.ncbi.nlm.nih.gov/gene/18773156, accessed on 16 May 2024). The encoded protein has a coding sequence length of 354 base pairs, as observed in the electropherogram of the gene ligation vector (Figure 1a). PpSnRK1β3 is structurally comprised of three helices, three strands, and seven coils, indicating a complex and intricate composition (Figure 1b). In addition, a subcellular localization fluorescence analysis demonstrated that it is present in both the nucleus and cell membrane (Figure 1c).

### 3.2. Phylogenetic Tree Analysis of PpSnRK1β3

**Figure 2:**
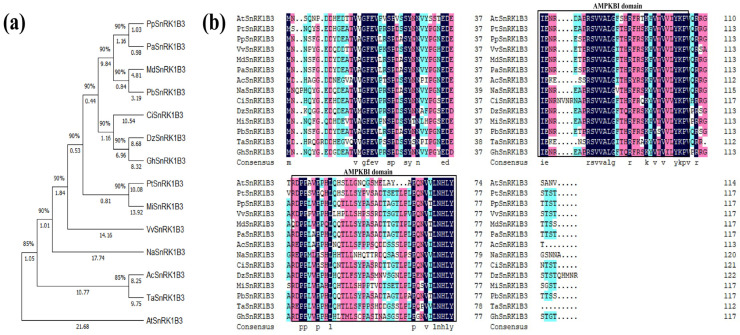
Phylogenetic tree analysis of PpSnRK1β3. (**a**) Phylogenetic tree analysis of the SnRK1β3 protein in different species. (**b**) Sequence alignment of the SnRK1β3 protein from different species.

For phylogenetic tree construction, we selected PpSnRK1β3 along with several other plant-encoded proteins: AtSnRK1β3 (NP_001323590.1), PtSnRK1β3 (XP_006384933.1), PpSnRK1β3 (XP_006384933.1), VvSnRK1β3 (XP_019076263.1), MdSnRK1β3 (XP_008359553.1), PaSnRK1β3 (XP_021812278.1), AcSnRK1β3 (XP_020085356.1), NaSnRK1β3 (XP_019255734.1), CiSnRK1β3 (XP_042955180.1), MiSnRK1β3 (XP_044506124.1), DzSnRK1β3 (XP_022732265.1), PbSnRK1β3 (XP_048444836.1), TaSnRK1β3 (XP_044447578.1), and GhSnRK1β3 (XP_016748682.2). Phylogenetic analysis revealed that PpSnRK1β3 is most closely related to PaSnRK1β3, followed by MdSnRK1β3 and PbSnRK1β3. Interestingly, the tree showed that PpSnRK1β3 is evolutionarily linked with fruit trees, whereas it is more distantly related to wheat, cotton, and *Arabidopsis* (Figure 2a). Multiple sequence alignment demonstrated conserved regions among the SnRK1β3 subunits from different plants. The NCBI domain annotation indicated the presence of AMPKBI (ASC) domains in all sequences (Figure 2b).

### 3.3. Relationship Between PpSnRK1β3 and the Functional Subunit PpSnRK1α

**Figure 3:**
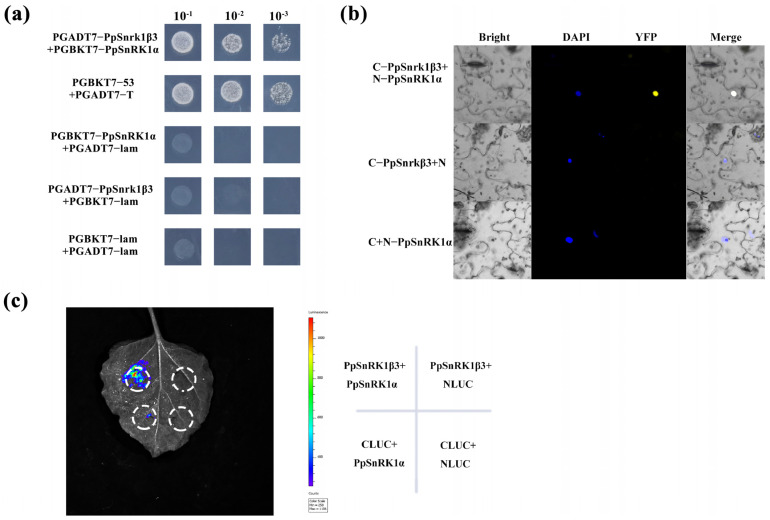
Interaction between the PpSnRK1β3 and PpSnRK1α subunits. (**a**) Yeast two-hybrid assay of PpSnRK1β3 and PpSnRK1α. (**b**) Bimolecular fluorescence complementation (BiFC) assay of PpSnRK1β3 and PpSnRK1α. (**c**) Dual luciferase assay of PpSnRK1β3 and PpSnRK1α.

The α core subunit of SnRK1 in plants plays an important role in the SnRK1 complex by catalyzing key reactions and containing conserved phosphorylation sites. This subunit is highly conserved across various plant species and is targeted by specific upstream kinases for phosphorylation [50,51]. In a yeast double hybrid assay, an interaction between the PpSnRK1β3 and PpSnRK1α subunits was observed. When AD-PpSnRK1β3 and BD-PpSnRK1α were co-transformed into yeast-deficient medium, the yeast was able to grow normally even after a 10^−3^ dilution. The transformed yeast exhibited similar growth patterns to the positive control, indicating a successful interaction between the subunits. In contrast, yeast transformed with empty vectors showed no growth on the deficient medium (Figure 3a).

After 2 days of incubation in tobacco, the Bimolecular Fluorescence Complementation assay (BiFC) of PpSnRK1α-pSPYNE and PpSnRK1β3-pSPYCE displayed a yellow fluorescent signal (YFP) in the tobacco nucleus. Furthermore, the YFP signal in the epidermis coincided with the blue fluorescent signal in the nucleus. These results suggest that the two subunits are interacting and bound together within the nucleus (Figure 3b).

After injecting PpSnRK1α-CLUC and PpSnRK1β3-NLUC into tobacco epidermal cells, fluorescence was observed three days later, demonstrating the interaction between PpSnRK1α and PpSnRK1β3 (Figure 3c).

### 3.4. GO Functional Analysis of DEGs Comparing OEPpSnRK1β3 Tomato and Wild-Type Tomato

The role of PpSnRK1β3 in tomato plants was explored by introducing the coding sequences that encode protein into stem segments infected with the Pri-101 vector. This resulted in the creation of primary transgenic tomato plants overexpressing PpSnRK1β3 (OEPpSnRK1β3) (Figure S1a). Among the transgenic plants, those showing high levels of expression were selected for further study. The selected plants were designated as T0-generation OEβ3-1, OEβ3-2, and OEβ3-3 (Figure S1b). Genetic screening of the T2 generation resulted in stable overexpression of PpSnRK1β3 in tomatoes, as shown in Figure S1c. Figure S1d revealed that there was no significant difference in the expression of transgene (PpSnRK1β3) in transgenic tomato plants.

The transcriptomes of OEPpSnRK1β3 tomatoes were compared with the wild-type (WT) during the same period by DNA sequencing. The samples generated between 25 and 33 million pair-end reads, resulting in a total of 7.6–9.9 billion clean base pairs. These sequences showed a consistent 42% G and C base composition, with a quality score of over 98.8% at Q20 and over 97% at Q30, which indicates a low base recognition error rate and high data quality overall. Alignment of the sample reads to the reference genome was achieved with >96% efficiency, which demonstrates a high alignment rate (Table S1). The raw sequencing data were deposited in the NCBI database under project number PRJNA1132511 for public access and further analysis (https://www.ncbi.nlm.nih.gov/bioproject/PRJNA1132511, accessed on 6 July 2024).

A Pearson correlation analysis for each of the three biological replicates demonstrated a strong relationship above 0.93, which meets the statistical criteria (Figure S2a). A comprehensive analysis yielded 18,968 differentially expressed genes (DEGs), characterized by |log2(FPKM) ratio| ≥ 1 and q value ≤ 0.05. A volcano plot revealed significant upregulation of 1156 DEGs and downregulation of 983 DEGs in the WT compared with OEPpSnRK1β3 tomatoes (Figure S2b). These findings highlight the robust reproducibility and significant gene expression differences between the two tomato varieties.

The GO database revealed a significant enrichment of differentially expressed genes (DEGs) in various categories for the WT samples compared with the PpSnRK1β3 tomatoes. The GO database revealed a significant enrichment of differentially expressed genes (DEGs) in various categories for WT samples compared to PpSnRK1β3 tomato. Downregulated WT DEGs were associated with cellular components (2349 times), molecular functions (959 times), and biological processes (2158 times) (Figure S3a). Conversely, the upregulated WT DEGs were associated with cellular components (2384 times), molecular functions (1090 times), and biological processes (2244 times) (the same gene would be annotated to different GO terms for multiple times) (Figure S3b). These findings highlight the diverse impact of the PpSnRK1β3 tomatoes on the gene expression profiles, suggesting a potential regulatory role in various cellular processes.

**Figure 4:**
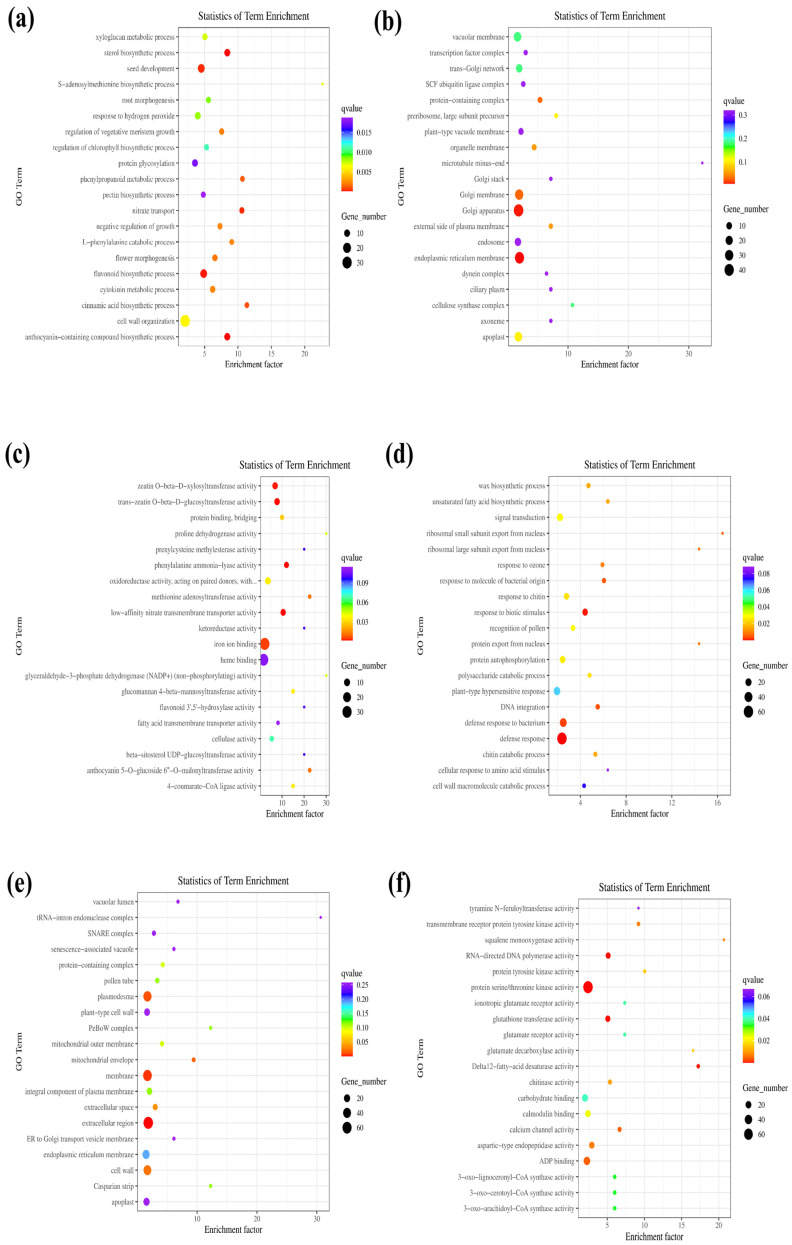
GO enrichment analysis of the comparison between OEPpSnRK1β3 and WT tomatoes. DEGs were selected based on a cut-off of *p*-adjust  <  0.05 and |log2FC| ≥ 1, *p*-adjust lists the top 20 enrichments in ascending order. (**a**–**c**) Downregulated WT genes were associated with biological processes, cellular components, and molecular functions in GO enrichment compared with the OEPpSnRK1β3 tomatoes. (**d**–**f**) WT upregulated in biological processes, cellular components, and molecular functions in GO enrichment compared with the OEPpSnRK1β3 tomatoes.

A differential gene expression analysis between WT plants and OEPpSnRK1β3 mutants revealed significant differences in multiple biological processes, cell compositions, and molecular functions. The OEPpSnRK1β3 mutants showed a marked upregulation in sterol biosynthesis, seed development, nitric acid transport, flavonoid biosynthesis, and anthocyanin-containing compounds compared with the WT plants (Figure 4a). In terms of cell composition, the OEPpSnRK1β3 mutants exhibited significant upregulation in the Golgi apparatus and endoplasmic reticulum membranes (Figure 4b). With respect to molecular function, the OEPpSnRK1β3 mutants exhibited higher expression levels of key enzymes such as zeatin xylosyltransferase, trans-corn glucosyltransferase, phenylalanine ammonia-lyase, and low-affinity nitrate transmembrane transporter (Figure 4c). In addition, a comparison between WT and OEPpSnRK1β3 tomato materials showed that WT plants were more sensitive to biostimuli during normal growth states, while WT was more sensitive to pollen recognition, ribosome export from the nucleus, and higher response to ozone than overexpressed materials (Figure 4d). With respect to cell composition, the OEPpSnRK1β3 mutants exhibited downregulation of plasmodesmata, membrane components, and extracellular space (Figure 4e). At the molecular function level, the OEPpSnRK1β3 mutants exhibited reduced activity in RNA-directed DNA polymerases, protein serine/threonine kinases, glutathione transferases, and amino acid kinases (Figure 4f).

### 3.5. KEGG Enrichment Analysis of DEGs

**Figure 5:**
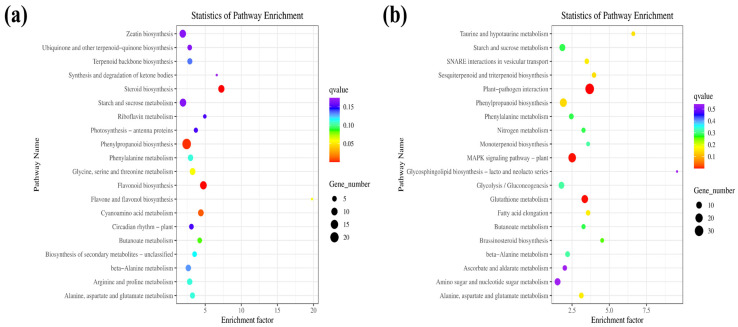
KEGG enrichment analysis of the comparison between OEPpSnRK1β3 and WT tomatoes. DEGs were selected based on a cut-off of *p*-adjust  <  0.05 and |log2FC| ≥ 1; *p*-adjust lists the top 20 enrichments in ascending order. (**a**) Downregulated WT genes by KEGG enrichment compared with OEPpSnRK1β3 tomatoes. (**b**) Upregulated WT genes by KEGG enrichment compared with OEPpSnRK1β3 tomatoes.

KEGG enrichment was used to identify DEG-related pathways [52]. This analysis revealed a significant upregulation of various pathways in both the WT and OEPpSnRK1β3 samples. In the OEPpSnRK1β3 mutants, pathways such as steroid biosynthesis, flavonoid biosynthesis, phenylpropanoid biosynthesis, and cyanamide acid metabolism were enriched (Figure 5a). Conversely, the OEPpSnRK1β3 sample exhibited significant downregulation in pathways associated with plant-pathogen interactions, MAPK signaling, glutathione metabolism, as well as several amino acid metabolism pathways (Figure 5b).

### 3.6. Effect of PpSnRK1β3 Overexpression on Tomato Growth

**Figure 6:**
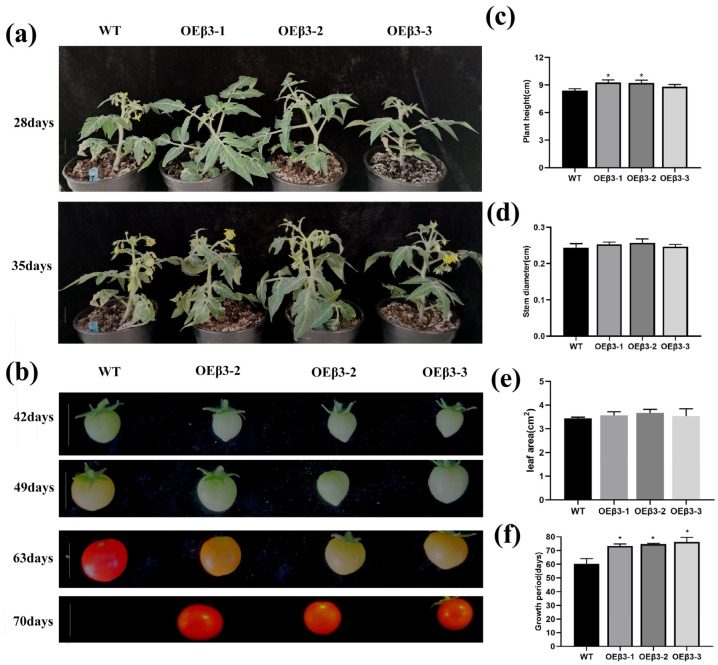
Growth characteristics of the OEPpSnRK1β3 tomatoes. (**a**) Growth period of OEPpSnRK1β3 and WT tomatoes (white line in the diagram indicates the height of 1 cm). (**b**) Fruit development period of OEPpSnRK1β3 and WT tomatoes (white line in the diagram indicates the length of 1 cm). Comparison of plant height (**c**), stem diameter (**d**), leaf area (**e**), and number of days in the growth period (**f**) for three strains of OEPpSnRK1β3 and WT tomatoes. Error bars represent the means ± SD (*n* = 3) from three independent biological replicates. Note: For (**c**–**f**), asterisks represent significant differences (LSD test, *, *p* < 0.05).

Tomatoes overexpressing PpSnRK1β3 exhibited a noticeable growth difference. These genetically modified tomatoes exhibited a delayed flowering period, which occurred 1–1.5 weeks later compared with WT tomatoes (Figure 6a). In addition, the ripening process of the fruit was also delayed, taking 1–2 weeks longer to reach maturity (Figure 6b).

The genetically modified β3 tomatoes exhibited robust growth, with mature plants reaching a slightly greater height compared with WT tomatoes (Figure 6c). Compared to WT tomatoes, transgenic tomatoes had a wider stem base width, but did not reach significant levels (Figure 6d). Moreover, there was no notable variance in leaf area between the OEPpSnRK1β3 tomatoes and the WT tomatoes (Figure 6e). Interestingly, the transgenic tomatoes did not display any signs of growth inhibition; however, they exhibited a longer vegetative period, taking 68–75 days from seed germination to fruit ripening, which was notably longer compared with the growth cycle of the WT tomatoes in a 16 h sunlight environment (Figure 6f). This suggests that the overexpression of the β3 gene in tomatoes may enhance growth without delaying development.

### 3.7. Effect of OEPpSnRK1β3 on Photosynthesis

**Figure 7:**
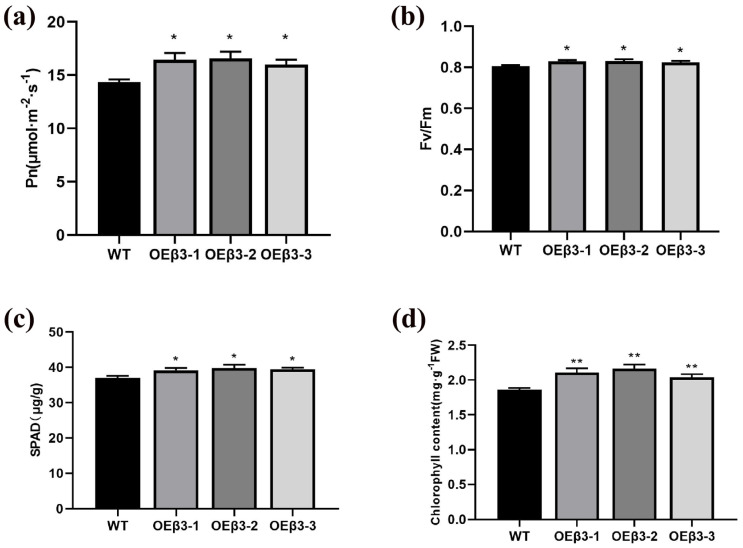
Photosynthetic indicators of OEPpSnRK1β3 tomatoes. Comparison of maximum net photosynthetic efficiency (**a**), chlorophyll content (**b**), stomatal conductance (**c**), and intercellular carbon dioxide concentration (**d**) for three strains of OEPpSnRK1β3 and WT tomatoes. Error bars represent the means ± SD (*n* = 3) from three independent biological replicates. Asterisks represent significant differences (LSD test, *, *p* < 0.05; **, *p* < 0.01).

The net photosynthetic rate (Pn) and chlorophyll fluorescence parameters (Fv/Fm) ratio of plants are important factors for establishing their photosynthetic capabilities. Higher Pn and Fv/Fm values indicate stronger photosynthetic capacity [53,54]. The SPAD value, which reflects the relative chlorophyll content in leaves, plays a key role in assessing photosynthetic efficiency [55]. OEPpSnRK1β3 tomatoes exhibited a significant increase in net photosynthetic efficiency and Fv/Fm compared with WT tomatoes (Figure 7a,b). In addition, the SPAD value and chlorophyll content of overexpressed tomatoes were higher than the WT tomatoes, which indicates that OEPpSnRK1β3 tomatoes enhance chlorophyll accumulation and improve photosynthetic activity (Figure 7c,d). Based on these findings, it is evident that PpSnRK1β3 overexpression in tomatoes positively influences photosynthetic activity.

### 3.8. Adaptability of OEPpSnRK1β3 Tomato to Drought Stress

**Figure 8:**
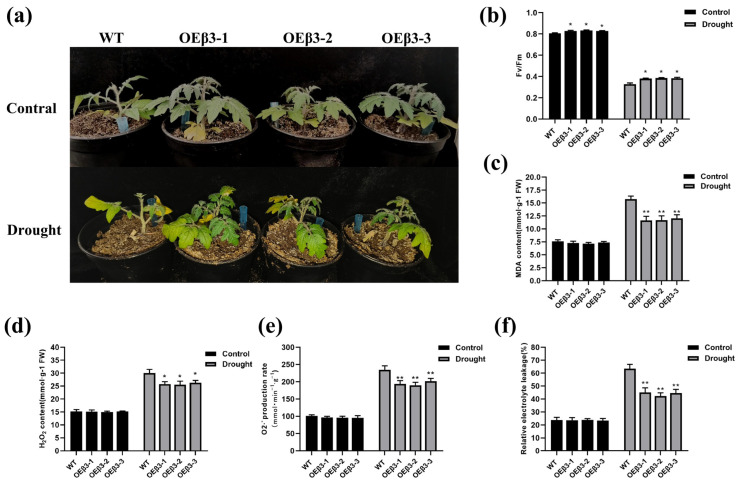
Physiological indexes of stress in OEPpSnRK1β3 tomatoes under drought stress. (**a**) The state of OEPpSnRK1β3 and WT tomatoes under normal and 14-day drought stress. Comparison of maximum photochemical efficiency (**b**), malondialdehyde content (**c**), hydrogen peroxide content (**d**), superoxide anion content (**e**), and relative electrolyte leakage (**f**) for three strains of OEPpSnRK1β3 and WT tomatoes under normal and 14-day drought stress conditions. Error bars represent the means ± SD (*n* = 3) from three independent biological replicates. Note: For (**b**–**f**), asterisks represent significant differences (LSD test, *, *p* < 0.05; **, *p* < 0.01).

After subjecting OEPpSnRK1β3 tomatoes to a 14-day simulated drought stress using 4% polyethylene glycol (PEG-6000), the leaves still retained some green color and the stems were able to maintain their normal life activities (Figure 8a). When assessing the photosynthetic efficiency and light capacity of leaves under stress, the chlorophyll fluorescence parameter ratio (Fv/Fm) is an essential indicator [56]. Following drought stress, the Fv/Fm ratio of the tomatoes decreased; however, the decrease was less significant in OEPpSnRK1β3 tomatoes compared with WT tomatoes, which indicates a superior photosynthetic capacity in the transgenic variety (Figure 8b). Furthermore, drought stress results in an increase in malondialdehyde (MDA) content in tomatoes, which causes peroxidation damage to the plant membrane lipids [57]. The MDA content in OEPpSnRK1β3 tomatoes was markedly lower compared with that in WT tomatoes. This suggests that the transgenic tomatoes have a protective effect on plant membrane lipids under drought conditions (Figure 8c). Under stress, the accumulation of reactive oxygen species in the body intensifies, resulting in damage to the cell membrane [58]. OEPpSnRK1β3 tomatoes exhibit a stronger ability to scavenge oxygen free radicals, which produces lower levels of superoxide anion and hydrogen peroxide under drought stress compared with WT tomatoes (Figure 8d,e). Under stressful conditions, plant cell membranes often suffer damage, resulting in an increase in relative electrolyte leakage [59]. Notably, the relative electrolyte leakage in transgenic tomatoes was significantly lower compared with that in WT tomatoes during drought stress, which highlights the enhanced drought resistance of the transgenic plants (Figure 8f).

## 4. Discussion

PpSnRK1β3 is a key component of the PpSnRK1 complex. It is unique compared with other β subunits because of its distinct lack of a GBD domain [9,19]. The GBD domain contains a phosphorylation site that enables interaction with proteins other than SnRK1 complex subunits [1]. In contrast, the SnRK1βγ subunit, which contains a GBD domain, regulates plant disease resistance by interacting with specific proteins associated with plant pathogen resistance [60]. This stark contrast suggests that subunits with the GBD domain can autonomously regulate plant functions. In the case of PpSnRK1β3, its effect on plants appears to be indirect, working in tandem with other SnRK1 subunits within the complex. Studies on the subcellular localization of the SnRK1 subunit in plants, such as *A. thaliana* and sorghum, indicate that the β3 subunit tends to concentrate in the cell membrane [23]. This was further confirmed by observing GFP fluorescence within the cell membrane of tobacco cells (Figure 1c). Interestingly, PpSnRK1β3 has also been detected in the nucleus, which suggests its potential role in linking various subunits of the SnRK1 complex. While the SnRK1β1 and β2 subunits contain a unique N-terminal myristoylation site capable of promoting the nucleation of the SnRK1α subunit under external stimuli, SnRK1β3 lacks this site [61]. Consequently, its absence may limit the effect of SnRK1β3 on the regulation of SnRK1α nucleation processes.

The presence of the SnRK1β3 subunit has been documented in various plant species such as *A. thaliana*, *Solanum lycopersicum*, and *Sorghum bicolor* [62,63,64]. Analysis of the proteins through sequence alignment and phylogenetic tree construction revealed that many plants contain the SnRK1β3 subunit with similar coding sequences. The phylogenetic tree of SnRK1β3 revealed that peach shares a closer homology with woody plants than herbaceous plants (Figure 2a). Various species of SnRK1β3 exhibit similar domains and potentially interact with similar proteins. In sorghum, the SnRK1 complex follows a polymeric pattern of α-β3-βγ subunits [62], whereas in peach, there is an interaction between SnRK1α and SnRK1β3 within the complex (Figure 3a–c). This interaction suggests that SnRK1β3 forms a complex with the catalytic subunit SnRK1α, which affects downstream reactions.

SnRK1, a key player in plant disease resistance, shows significant interactions with WRKY3, which is a suppressor of fungal resistance. By phosphorylating and destabilizing WRKY3, SnRK1 enhances the immunity of barley against powdery mildew disease [63]. OEPpSnRK1β3 results in the downregulation of genes associated with disease resistance and defense response (Figure 4d), which indicates that PpSnRK1β3 is related to plant biological defense, but under normal growth, SnRK1 can maintain the balance between growth and defense mechanisms [65]. This may be due to an adaptation of the plant to promote growth in a favorable environment at the expense of resistance gene expression. In addition to its role in disease resistance, SnRK1 also regulates amino acid content and the tricarboxylic acid cycle by phosphorylating bZIP proteins to counteract energy stress caused by darkness [66]. Notably, OEPpSnRK1 in tomatoes significantly regulates the activity of amino acid kinases, indicating that it regulates amino acid synthesis and metabolism (Figure 4c,f). Furthermore, plants can accumulate flavonoids with antioxidant properties [67]. Increased levels of flavonoid synthesis genes were observed in tomato leaves with overexpression of PpSnRK1 (Figure 4a). SnRK1 kinase can stably accumulate anthocyanins under long-term light conditions, which is beneficial for the photoprotection of plants [68]. Anthocyanin synthesis is a subset of flavonoid biosynthesis, which sheds light on the increased expression of flavonoid synthesis genes in plants with PpSnRK1β3 overexpression (Figure 4a). This suggests a link between SnRK1 activity and the regulation of flavonoid biosynthesis pathways in plants.

The phenylpropanoid metabolism pathway in plants is widely involved in lignification and nodule formation, enhancing plant resistance [69,70]. A KEGG pathway analysis revealed that overexpressing PpSnRK1β3 genes results in the enrichment of phenylpropanoid metabolism pathway genes (Figure 5a), thereby showcasing its potential for enhancing plant resistance. In addition, the role of the flavonoid biosynthesis pathway in enhancing plant resistance to stress has been reported [71,72]. We found that OEPpSnRK1β3 DEGs were also enriched in the flavonoid biosynthesis pathway, indicating an improvement in the plant’s ability to improve plant resistance (Figure 5a). Furthermore, the enrichment pathway diagram illustrates the effect of PpSnRK1β3 on tomato sugar and starch metabolism pathways, emphasizing its role in regulating glucose metabolism to maintain overall plant health and resilience [73].

The role of SnRK1α in plant growth and energy maintenance is important, particularly during times of low energy availability [6,74]. SnRK1α inhibits plant growth to conserve energy and ensure survival [75]. On the other hand, OEPpSnRK1β3 cannot hinder plant growth and stem thickening under normal conditions (Figure 6c,d). This may occur because the inhibition of plant growth by SnRK1 cannot be stimulated under normal conditions. The activity of the SnRK1 enzyme in tomato leaves overexpressing PpSnRK1α results in increased net photosynthetic rates, which indicates a positive effect on plant growth [74]. Moreover, PpSnRK1β3 promotes chlorophyll synthesis and enhances photosynthetic efficiency (Figure 7), which leads to continuous plant growth and delayed flowering (Figure 6a,f). There was a similar phenotype between delayed flowering and SnRK1α in PpSnRK1β3 under long sunlight [60]. Furthermore, the SnRK1β subunit may independently regulate the plant stress response and enhance drought resistance by improving defense mechanisms against stress factors [76]. The PpSnRK1β3 subunit promotes the drought resistance of plants (Figure 8), which is associated with the improvement of defense against stress by the β subunit. However, the precise interaction between PpSnRK1β3 and PpSnRK1α under stress conditions remains unclear; thus, further study on the regulatory effects of PpSnRK1β3 on the core subunits is needed. This emphasizes the complexity and importance of SnRK1 subunits in plant growth, energy maintenance, and stress response mechanisms. In the functional verification of the SnRK1β3 subunit, there is no report on the identification of mutant function, which is the factor restricting the functional verification of SnRK1β3, which is also a part worthy of attention and exploration in the study of the SnRK1 complex.

## 5. Conclusions

In this study, PpSnRK1β3 was analyzed as a single gene. PpSnRK1β3 has a cds of 354 bp. and is composed of helices, strands, and coils. It is primarily localized to the nucleus and cell membrane. PpSnRK1β3 interacts with the PpSnRK1α subunit. Genes that are associated with the overexpression of PpSnRK1β3 in tomatoes include sterol biosynthesis, seed development, nitric acid transport, and flavonoid biosynthesis pathway genes. The growth period for tomatoes overexpressing PpSnRK1β3 was prolonged, and the characteristics of vigorous growth were evident. Tomatoes overexpressing PpSnRK1β3 showed an increase in photosynthesis activity and strong drought resistance. 

## Data Availability

Data are contained in the article and Supplementary Materials.

## Supplementary Materials

The following supporting information can be downloaded at https://www.mdpi.com/article/10.3390/genes15121574/s1. Figure S1: Acquisition of PpSnRK1β3 overexpressing tomatoes. (a) Growth of PpSnRK1β3-infected tomatoes in symbiosis medium, differentiation medium, and rooting medium. (b) Identification and screening of the T0 generation of PpSnRK1β3 expressing tomatoes. (c) Identification and screening of the T2 generations of PpSnRK1β3 expressing tomatoes. (d) Quantitative fluorescence PCR detection of PpSnRK1β3 gene expression in T2-generation tomatoes; Figure S2: DEGs analysis of WT vs. OEPpSnRK1β3 tomatoes. (a) Pearson correlation analysis of the DEGs in WT vs. OEPpSnRK1β3 tomatoes. (b) DEG volcanogram analysis of WT vs. OEPpSnRK1β3 tomatoes; Figure S3: GO enrichment analysis of WT vs. OEPpSnRK1β3 tomatoes. (a) WT vs. OEPpSnRK1β3 tomatoes significantly downregulated DEGs from a GO enrichment analysis. (b) WT vs. OEPpSnRK1β3 tomatoes significantly upregulated DEGs in GO enrichment analysis. Table S1: Summary of RNA-Seq reads. Table S2: Primer sequences used in this article.

## Abbreviations

SnRK1	Sucrose Non-fermentation-related Kinase 1
OE	Overexpression
Pp	*Prunus persica*
BiFC	Bimolecular Fluorescence Complementation
CBM	Carbohydrate-binding module
DEG	Differentially expressed genes
FLZ	FCS-like zinc
GO	Gene Ontology
KEGG	Kyoto Encyclopedia of Genes and Genomes
MAPK	Mitogen-activated protein kinase
NCBI	National Center for Biotechnology Information
TBA	Thiobarbituric acid
TCA	Trichloroacetic acid
WT	Wild-type
