# Supplementary material for: Genetic Analysis of the Peach SnRK1β3 Subunit and Its Function in Transgenic Tomato Plants
Source: Genes (Basel). 2024 Dec 6;15(12):1574. doi: 10.3390/genes15121574 (PMC11675834; doi:10.3390/genes15121574)
Supplement: Supplementary file 1 [file genes-15-01574-s001.zip › S table.pdf]

Table S1. Summary of RNA-Seq reads

Sample	ReadSum	BaseSum	GC(%)	N(%)	Q20(%)	Q30(%)	reads aligned
OEB3-1	27898185	8369455500	42.9	0.01	98.86	97.09	53777222(96.38%)
OEB3-2	27790384	8337115200	42.97	0.01	98.85	97.05	53665473(96.55%)
OEB3-3	25567375	7670212500	42.91	0.01	98.85	97.03	49339386(96.49%)
WT1	28191389	8457416700	42.63	0.01	98.85	97.05	54120713(95.99%)
WT2	25982296	7794688800	42.98	0.01	98.83	97	49885413(96.00%)
WT3	32841073	9852321900	42.94	0.01	98.81	97	63122707(96.10%)

Table S2. Primer sequences used in this article

Primer name	Use	Sequence
Pri101β3-F	Construction of infected tomato vector, RT-PCR, Subcellular localization	ttgatacatatgcccgtcgacATGAACAACTCATTTGGTGATGATTA
Pri101β3-R		tttacccatgaattcggatccAGTGCTGGTAGTCCCCCTTC
PGADT7β3-F	Yeast two-hybrid	gccatggaggccagtgaattcATGAACAACTCATTTGGTGATGATTA
PGADT7β3-R		cagctcgagctcgatggatccCTAAGTGCTGGTAGTCCCCCTTC
PGBKT7α-F		atggccatggaggccgaattcATGGATGGATCGGTTGGCC
PGBKT7α-R		ccgctgcaggtcgacggatccTTAAAGGACCCGAAGTTGTGCA
YCβ3-F	BiFC	tggcgcgccactagtggatccATGAACAACTCATTTGGTGATGATTA
YCβ3-R		agcggtaccctcgaggtcgacCTAAGTGCTGGTAGTCCCCCTTC
YNα-F		tggcgcgccactagtggatccATGGATGGATCGGTTGGCC
YNα-R		agcggtaccctcgaggtcgacTTAAAGGACCCGAAGTTGTGCA
NLUCβ3-F	Luci-ferase complementation	cgagctcggtacccgggatccATGAACAACTCATTTGGTGATGATTA
NLUCβ3-R		cgcgtacgagatctggtcgacCTAAGTGCTGGTAGTCCCCCTTC
CLUCα-F		ccggggcggtacccgggatccATGGATGGATCGGTTGGCC
CLUCα-R		acgaaagctctgcaggtcgacTTAAAGGACCCGAAGTTGTGCA
SlEF1α-F	qRT-PCR	TGGAAACGGATATGCCCCTG
SlEF1α-R		TGGGCTTGGTGGGAATCATC
PpSnRK1β3-F		CAACTGTTGCAGGATTTGAAGT
PpSnRK1β3-R		TGCTGGTAGTCCCCCTTCTT
