# Supplementary figures and images for: Genetic Analysis of the Peach SnRK1β3 Subunit and Its Function in Transgenic Tomato Plants
Source: Genes (Basel). 2024 Dec 6;15(12):1574. doi: 10.3390/genes15121574 (PMC11675834; doi:10.3390/genes15121574)

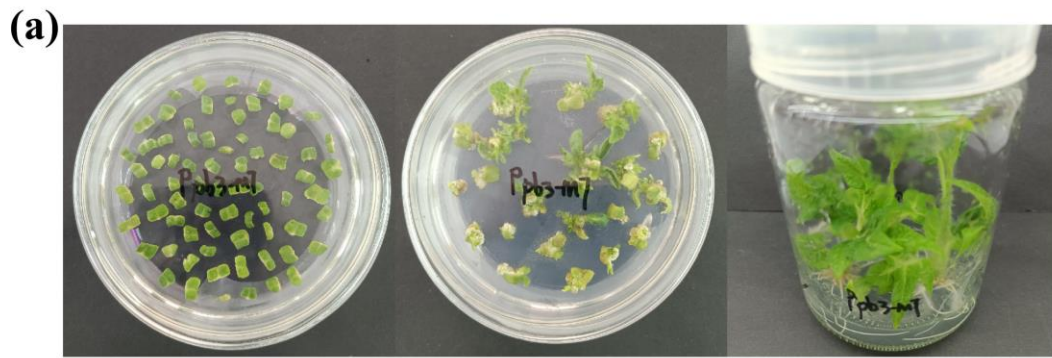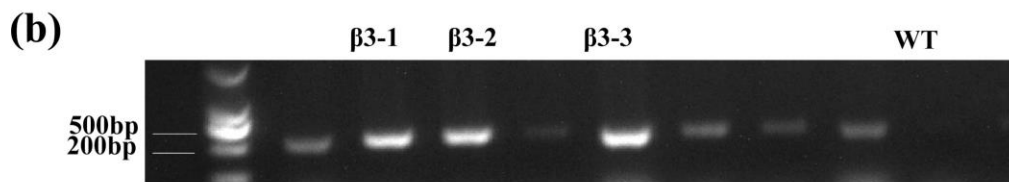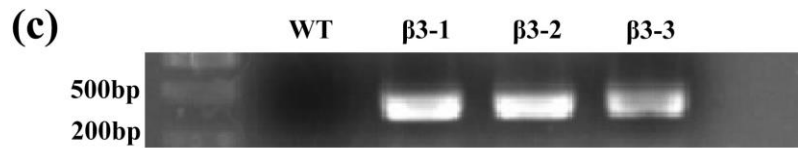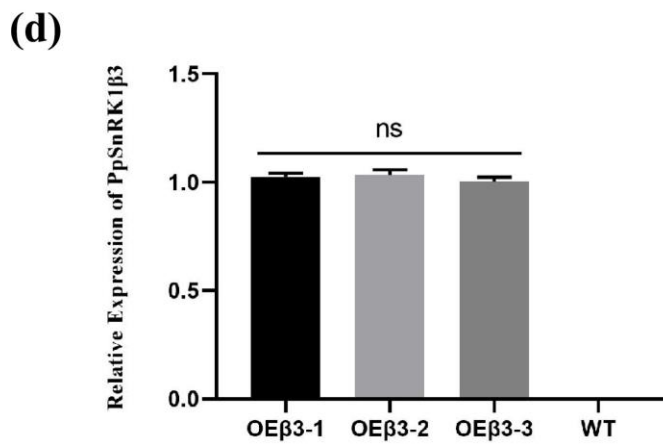

Supplement: Supplementary file 1 [file genes-15-01574-s001.zip › S1.pdf]

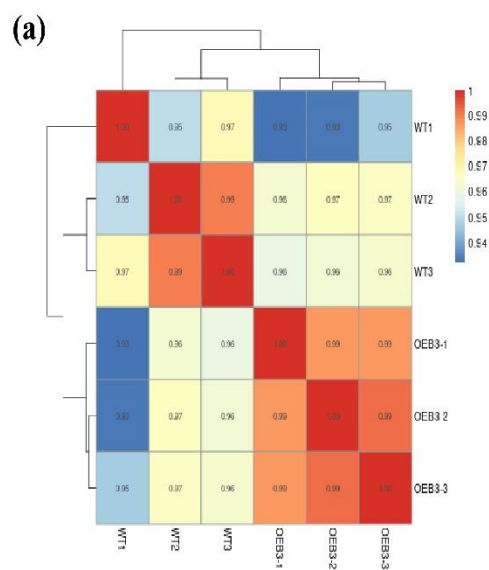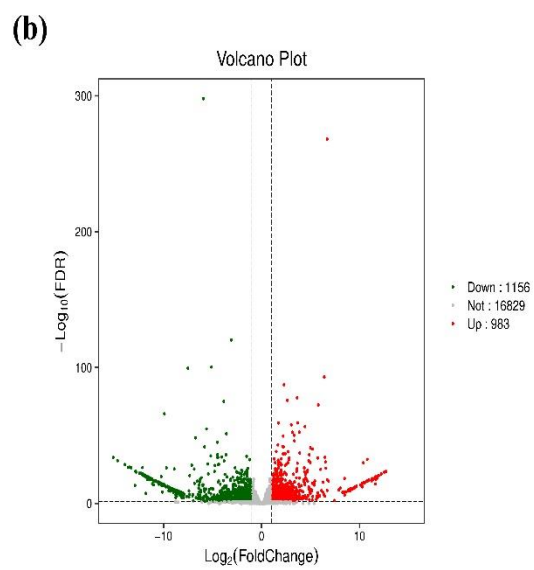

Supplement: Supplementary file 1 [file genes-15-01574-s001.zip › S2.pdf]

(a)

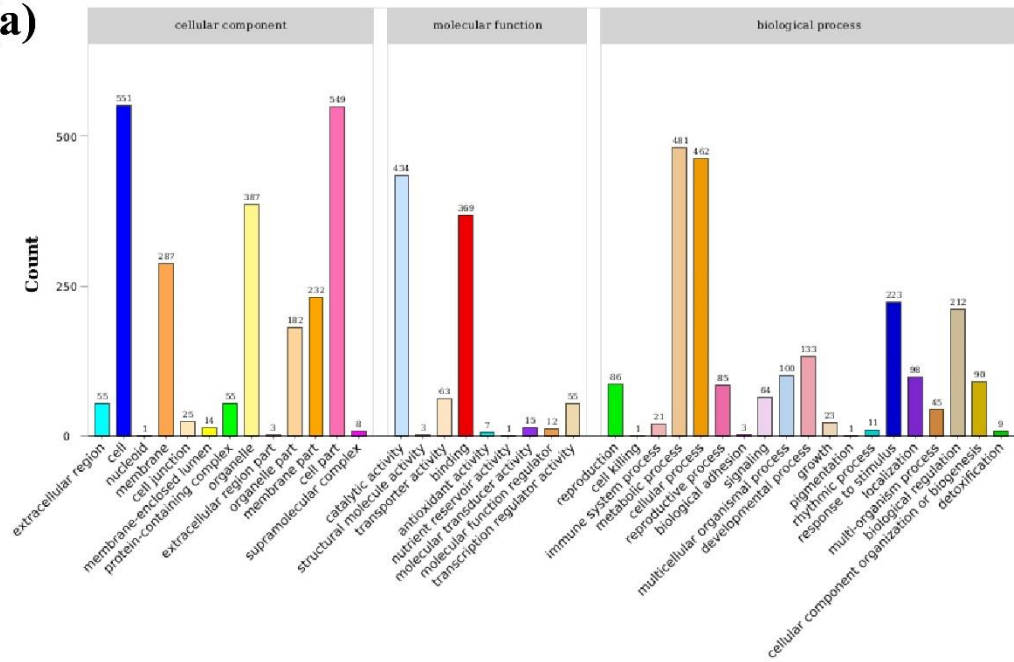

(b)

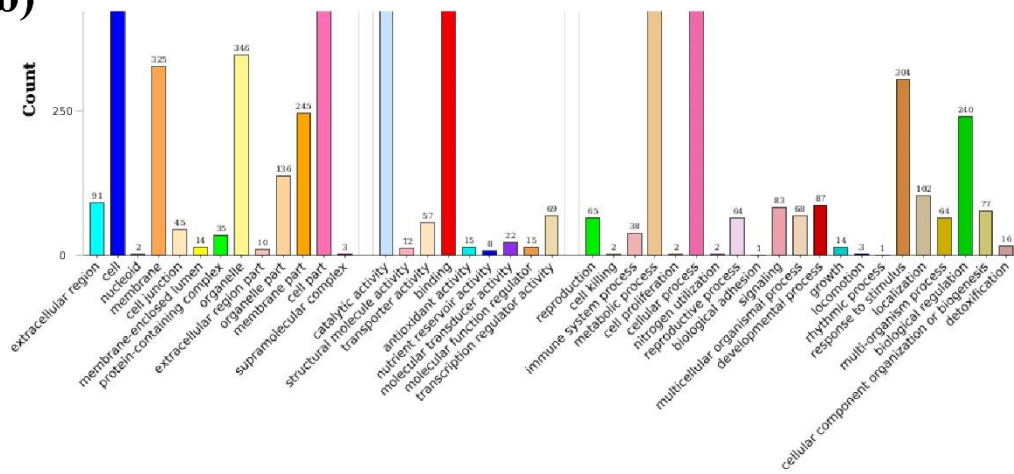

Supplement: Supplementary file 1 [file genes-15-01574-s001.zip › S3.pdf]
